# Anti-Inflammatory Activities of Baobab Fruit Extracts in TNF-*α*/IFN-*γ*-Stimulated HaCaT Keratinocytes with LC–MS/MS and HPLC Profiling

**DOI:** 10.3390/ph19040639

**Published:** 2026-04-18

**Authors:** Shi-Heon Kang, Soon Yeong Park, Hoon Kim, Sanghyun Lee

**Affiliations:** 1 Department of Plant Science and Technology, Chung-Ang University, Anseong 17546, Republic of Korea; 2 CNS Pharm Korea Co., Ltd., Seoul 04043, Republic of Korea; 3 Department of Food and Nutrition, Anyang University, Anyang 14028, Republic of Korea; 4 Natural Product Institute of Science and Technology, Anseong 17546, Republic of Korea

**Keywords:** baobab (*Adansonia digitata*), keratinocytes, TNF-*α*/IFN-*γ*, anti-inflammatory activity, LC–MS/MS, polyphenols

## Abstract

**Background/Objectives**: Atopic dermatitis (AD)-related skin inflammation involves the release of cytokines and chemokines from keratinocytes; therefore, keratinocyte-based models are widely used to evaluate the anti-inflammatory potential of botanical extracts. This study examined the relationship between phytochemical profiles and anti-inflammatory potential of baobab fruit 30% and 70% ethanol extracts (BE-30 and BE-70, respectively) in a TNF-*α*/IFN-*γ* (TI)-stimulated HaCaT keratinocyte model. **Methods**: The anti-inflammatory effects of both extracts were evaluated by measuring cytokine and chemokine secretion in TI-stimulated HaCaT cells. Phytochemical characterization was performed using liquid chromatography–tandem mass spectrometry (LC–MS/MS) and targeted high-performance liquid chromatography (HPLC). **Results**: Both extracts were non-cytotoxic. TI-stimulation markedly increased interleukin (IL)-6, IL-8 and monocyte chemotactic protein (MCP)-1 secretion, while BE-30 and BE-70 significantly reduced all three mediators in a dose-dependent manner. At comparable doses, BE-70 exhibited greater inhibition than BE-30. BE-30 showed a non-monotonic IL-8 response at low concentrations, whereas BE-70 consistently reduced IL-8 in a dose-dependent manner. LC–MS/MS profiling revealed a polyphenol-rich composition, including flavonol glycosides and related phenolic compounds. HPLC confirmed the presence of four marker analytes (procyanidin B2, epicatechin, rutin and tiliroside), which were enriched in BE-70. The content of these four polyphenols was 1.94-fold higher in BE-70. **Conclusions**: Baobab fruit extracts exhibit anti-inflammatory activity associated with polyphenols. These findings suggest that they could be used as analytical standards and in dermatological applications.

## 1. Introduction

Atopic dermatitis (AD) is a chronic, pruritic inflammatory skin disease affecting 2–10% of the adult population and up to 20% of children worldwide [1,2]. The pathophysiology of AD involves immune dysregulation, epidermal barrier dysfunction, and genetic predisposition [3,4]. Keratinocytes are immune-active epithelial cells in AD and related inflammatory dermatoses, producing cytokines and chemokines that amplify local inflammatory circuits and promote leukocyte recruitment [5]. Among these mediators, interleukin-8 (IL-8) and monocyte chemoattractant protein-1 (MCP-1) are key chemokines involved in leukocyte recruitment, whereas interleukin-6 (IL-6) is a representative keratinocyte-associated inflammatory cytokine [6]. Consistent with this rationale, tumor necrosis factor-*α* (TNF-*α*) and interferon-*γ* (IFN-*γ*) co-stimulation reproducibly elevates IL-6, IL-8, and MCP-1 secretion in HaCaT keratinocytes, providing an experimentally tractable platform for in vitro anti-inflammatory screening [7]. Accordingly, there is a continuing need for safer, natural product-derived candidates that can concurrently attenuate inflammation and reinforce epidermal barrier function [8,9].

Natural product-derived extracts remain important sources of bioactive compounds, but their biological relevance should be supported by clear phytochemical characterization [10,11,12]. In this context, establishing both chemical and biological evidence is essential for evaluating the therapeutic potential of selected botanical resources.

Baobab (*Adansonia digitata* L.) is a tree native to the African savanna, revered for its contributions to nutrition, medicine, and cultural practice [13]. Baobab fruit pulp is exceptionally rich in polyphenolic compounds, with procyanidin B2, epicatechin, rutin-type flavonol glycosides, and tiliroside among the major compounds reported in previous phytochemical studies [14,15]. Several baobab-derived constituents influence inflammatory and barrier-associated pathways relevant to AD. Quercetin-related flavonols have been associated with modulation of PPAR-*α*-related signaling, including effects on TLR4 and NF-*κ*B pathways and Th2-associated cytokine responses [8,16]. In addition, procyanidin B2 has been reported to attenuate inflammatory responses through reduction in oxidative stress and inhibition of IL-1*β* production [17]. Baobab fruit pulp is also a source of polyunsaturated fatty acids and vitamins associated with epidermal barrier support in inflammatory skin contexts [13,18]. Taken together, these observations provide a rationale for examining whether baobab fruit extracts can attenuate TNF-*α*/IFN-*γ* (TI)-driven keratinocyte inflammation.

Despite these reports, AD-focused evidence for baobab fruit extracts remains limited in the peer-reviewed literature. Baobab fruit extracts have rarely been evaluated in keratinocyte-based inflammatory systems used to model AD-relevant epithelial inflammation under TI co-stimulation. In addition, the quantitative association between polyphenols and keratinocyte cytokine responses remains insufficiently characterized. This gap limits mechanistic interpretation at the extract level and hinders the development of reproducible, analytically anchored evidence that can be compared across studies. Accordingly, the present study addresses this gap by evaluating baobab fruit extracts in TI-stimulated keratinocytes using AD-relevant inflammatory readouts [19,20,21,22].

This study was designed to characterize the phytochemical composition of baobab fruit extract using liquid chromatography-tandem mass spectrometry (LC-MS/MS) and high-performance liquid chromatography (HPLC). Quantitative polyphenol profiles were then interpreted in relation to these cytokine responses. The resulting data provide a reproducible reference framework for future studies of standardized baobab-derived preparations in dermatologic research.

## 2. Results and Discussion

### 2.1. Non-Cytotoxicity and Anti-Inflammatory Effects of Baobab Fruit Extracts

Baobab fruit powder was extracted separately with 30% and 70% ethanol (EtOH) to obtain two hydroethanolic preparations with different solvent polarities. These two extraction conditions were selected to compare relatively water-rich and ethanol-rich solvent environments, because the EtOH–water ratio is known to influence the recovery of different phytochemical classes [23]. In previous extraction studies, 30% aqueous EtOH has been associated with improved recovery of relatively polar constituents such as phenolic acids, whereas 70% aqueous EtOH has frequently been used to enhance recovery of broader phenolic constituents, including flavonoids [23]. After extraction, each filtrate was concentrated under reduced pressure to obtain the corresponding crude extract. The resulting 30% EtOH extract and 70% EtOH extract were designated as BE-30 and BE-70, respectively, and were used for the subsequent biological and phytochemical analyses.

**Figure 1:**
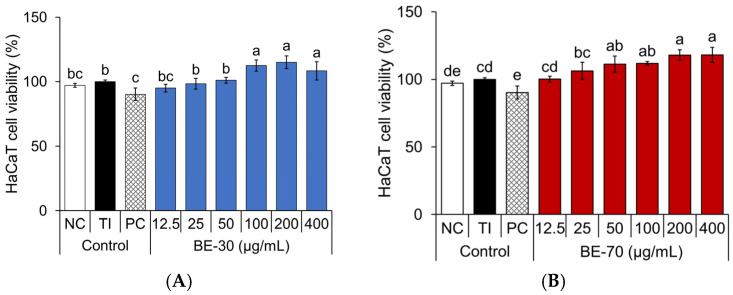
Effects of the BE-30 (**A**) and BE-70 (**B**) extracts on cell viability in TNF-*α*/IFN-*γ* (TI)-stimulated HaCaT cells. Negative control (NC) treated with 0.1% DMSO in serum-free Dulbecco’s modified Eagle’s medium (DMEM), and positive control (PC) treated with dexamethasone. Cell viability was measured using the MTT assay. Data are expressed as the mean ± standard deviation (SD) (*n* = 3). Different letters above the bars (a–e) denote statistically distinct groupings within each panel as determined by Tukey’s multiple-comparison test at *p* < 0.05; groups sharing a letter are not significantly different.

To evaluate the anti-inflammatory potential of baobab fruit extracts, experimental conditions without overt cytotoxicity were first established in TI-stimulated HaCaT keratinocytes [24]. TI-stimulation did not reduce the 3-(4,5-dimethylthiazol-2-yl)-2,5-diphenyltetrazolium bromide (MTT) signal relative to the negative control (NC), with the MTT signal remaining close to 100% of the NC level, indicating that the inflammatory condition used in this study was not associated with overt cytotoxicity (Figure 1). Under these conditions, BE-30 at 12.5–50 μg/mL and BE-70 at 12.5–25 μg/mL showed MTT signals broadly comparable to the TI control, while the upper concentration range of both extracts produced elevated MTT signals. As MTT reduction reflects cellular metabolic activity and does not directly distinguish changes in cell number from changes in cellular reduction capacity, the elevated signals were interpreted conservatively and were not considered sufficient to demonstrate enhanced proliferation [25]. Accordingly, these concentrations were carried forward to subsequent cytokine assays because no decrease in the MTT signal was observed under the present conditions. Additional proliferation-focused assays will be required to determine whether the elevated signals observed at the upper tested concentrations are associated with altered keratinocyte proliferation.

**Figure 2:**
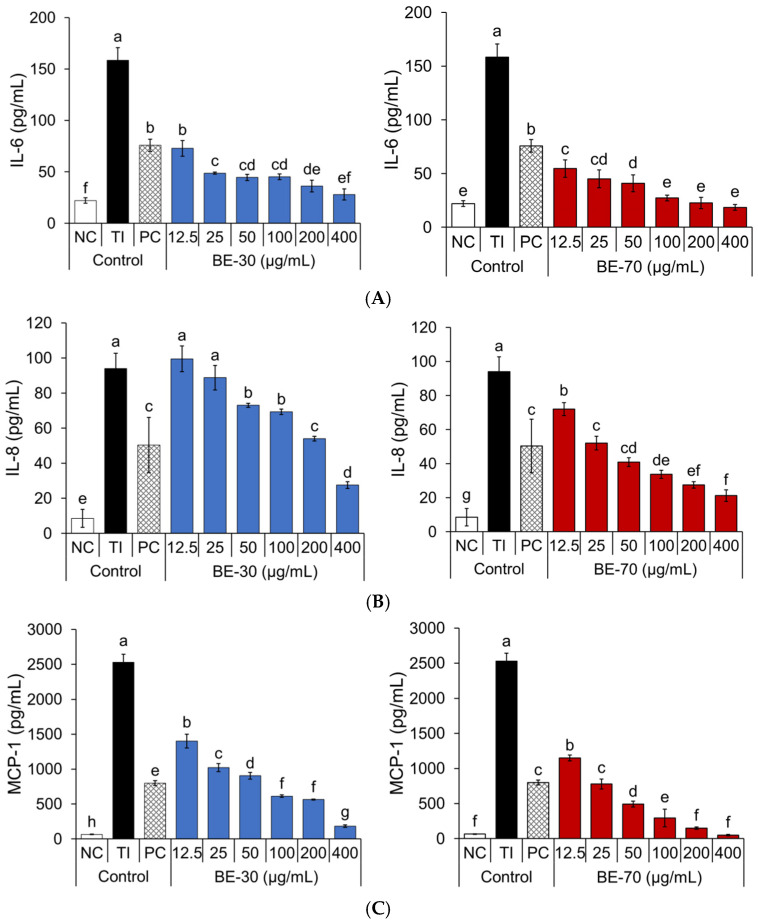
Effects of the BE-30 and BE-70 extracts on IL-6 (**A**), IL-8 (**B**), and MCP-1 (**C**) secretion in TNF-*α*/IFN-*γ* (TI)-stimulated HaCaT cells are demonstrated. Negative control (NC) treated with 0.1% DMSO in serum-free Dulbecco’s modified Eagle’s medium (DMEM), and positive control (PC) treated with dexamethasone. IL-6, IL-8, and MCP-1 levels in the culture supernatant are quantified by enzyme-linked immunosorbent assay (ELISA). Data are expressed as the mean ± standard deviation (SD) (*n* = 3). Different letters above the bars (a–h) denote statistically distinct groupings within each panel as determined by Tukey’s multiple-comparison test at *p* < 0.05; groups sharing a letter are not significantly different.

The effects of BE-30 and BE-70 on IL-6, IL-8, and MCP-1 secretion were evaluated in TI-stimulated HaCaT keratinocytes across the tested concentration range (Figure 2). TI-stimulation robustly increased IL-6, IL-8, and MCP-1 secretion in HaCaT cells, confirming strong inflammatory induction. Both baobab fruit extracts attenuated IL-6 in a dose-dependent manner, with BE-70 showing consistently stronger inhibition than BE-30 at matched doses. For IL-8, BE-30 exhibited a non-monotonic response, with minimal inhibition at the lowest concentrations and clearer reductions emerging only at higher concentrations, whereas BE-70 produced a stepwise decrease across the full concentration range, indicating a broader effective window for chemokine control. MCP-1, the most strongly induced mediator under TI-stimulation, was also reduced by both extracts in a dose-dependent fashion, with BE-70 achieving earlier and deeper inhibition than BE-30 as concentration increased. Taken together, the cytokine and chemokine profiles support a concentration-dependent anti-inflammatory trend for both extracts, while consistently indicating greater potency and a wider effective range for BE-70 in this TI-stimulated keratinocyte model.

The positive control (PC), dexamethasone, was applied at 20 µg/mL, and therefore focused on BE-30 and BE-70 at 12.5 and 25 µg/mL. In this range, BE-70 showed concurrent inhibition of IL-6, IL-8, and MCP-1. At 12.5 µg/mL, BE-70 showed higher IL-6 inhibition (76.1%) than the PC (60.5%). At 25 µg/mL, BE-70 maintained a stronger inhibitory effect on IL-6 (83.1%) than the PC (60.5%) and showed inhibition values close to the PC for IL-8 and MCP-1, with IL-8 inhibition of 49.1% for BE-70 and 51.0% for the PC, and MCP-1 inhibition of 71.0% for BE-70 and 70.1% for the PC. BE-30 at 12.5 to 25 µg/mL showed IL-6 inhibition of 62.8% and 80.5%, but IL-8 was not inhibited at 12.5 µg/mL and showed only 6.1% inhibition at 25 µg/mL, while MCP-1 inhibition was 45.7% and 61.2%, remaining below that of the PC (70.1%). Compared with BE-30, BE-70 provided earlier and broader inhibition across the three mediators.

Previous studies on baobab have predominantly used methanolic or hydroethanolic extracts of leaves or trunk bark, or crude fruit pulp and seed preparations, and have evaluated anti-inflammatory effects in systemic models or immune-cell–based assays such as LPS-stimulated RAW264.7 macrophages, often at relatively high doses [26,27,28]. In addition, topical application of baobab seed oil has been reported to be well tolerated on human skin while improving hydration and barrier function, supporting its potential as a dermocosmetic ingredient [29]. In contrast, the present study examines ethanol-fractionated baobab fruit extracts, BE-30 and BE-70, directly in a cytokine-driven human keratinocyte inflammation model, comparing them while simultaneously assessing IL-6, IL-8, and MCP-1, thereby providing novelty in both experimental model and dose range. Given prior reports that baobab leaf extracts can inhibit NF-*κ*B signaling, BE-30 and BE-70 may likewise act through NF-*κ*B and MAPK pathways. Although this study did not directly investigate these mechanisms, future research examining MAPK phosphorylation and NF-*κ*B nuclear translocation in TI-stimulated HaCaT cells, along with studies in 3D skin equivalents and in vivo dermatitis models, is needed to validate this hypothesis [26].

### 2.2. LC–MS/MS and HPLC Profiling Reveal a Polyphenol-Rich Basis for the Anti-Inflammatory Activity

**Figure 3:**
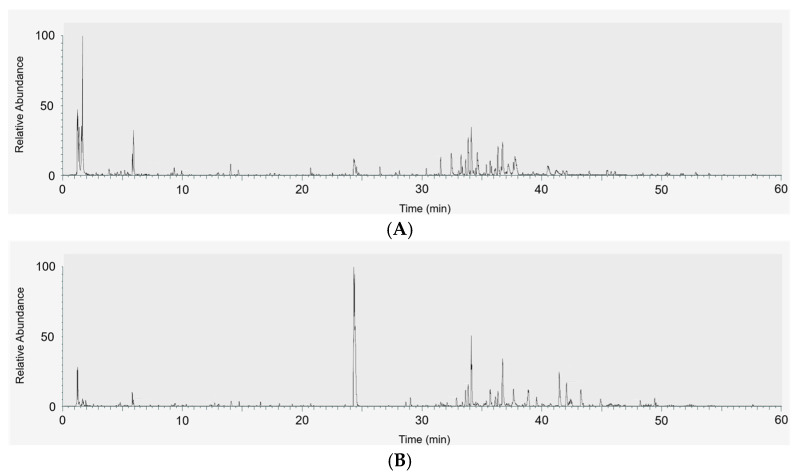
LC–MS/MS base-peak chromatograms of baobab fruit extracts in negative (**A**) and positive (**B**) ion modes.

To elucidate why baobab fruit extracts exhibit strong anti-inflammatory effects, and why BE-70 tends to be more potent than BE-30, detailed phytochemical profiling was performed using LC–MS/MS and HPLC. LC–MS/MS base-peak chromatograms of baobab fruit extracts in negative and positive ion modes revealed a chemically complex extract with numerous peaks distributed over the chromatogram, indicating the presence of multiple polarity metabolites (Figure 3).

Targeted MS analysis, summarized in Table 1 and Table 2, allowed tentative identification of 13 major ions based on precursor *m*/*z*, accurate mass, molecular formula, and characteristic fragment ions. The annotated constituents were broadly represented by flavonoids and related phenolic compounds, including flavonol glycosides, flavan-3-ols, phenolic acids, and acylated flavonoid derivatives, some of which correspond to compounds previously reported from baobab fruit [14]. To strengthen annotation confidence, LC–MS/MS features showing Δ*m*/*z* errors greater than 4 ppm were treated as lower confidence and were not prioritized for subsequent standard-based HPLC quantification, which was ultimately restricted to analytes exhibiting clear chromatographic peaks and acceptable calibration performance [30,31].

**Figure 4:**
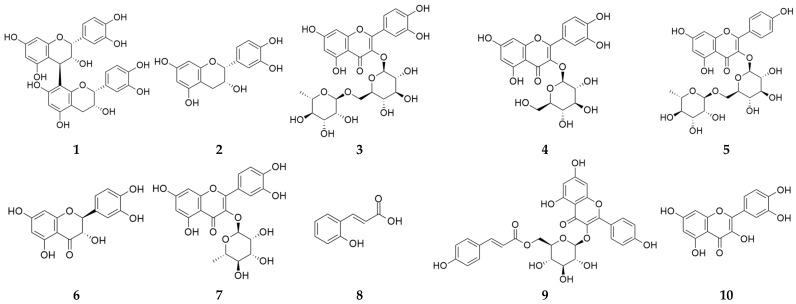
Chemical structures of procyanidin B2 (**1**), epicatechin (**2**), rutin (**3**), isoquercetin (**4**), nicotiflorin (**5**), taxifolin (**6**), quercitrin (**7**), *o*-coumaric acid (**8**), tiliroside (**9**), and quercetin (**10**).

Accordingly, ten compounds were selected for targeted HPLC quantification: procyanidin B2, epicatechin, rutin, isoquercetin, nicotiflorin, taxifolin, quercitrin, *o*-coumaric acid, tiliroside, and quercetin. The chemical structures of these reference compounds are shown in Figure 4.

**Figure 5:**
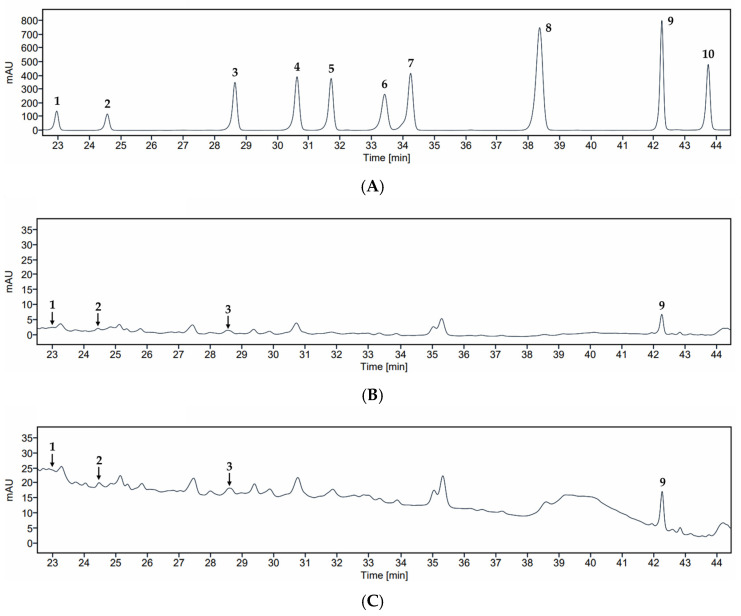
HPLC chromatograms of compounds **1**–**10** (**A**), BE-30 (**B**), and BE-70 (**C**). Compounds: procyanidin B2 (**1**), epicatechin (**2**), rutin (**3**), isoquercetin (**4**), nicotiflorin (**5**), taxifolin (**6**), quercitrin (**7**), *o*-coumaric acid (**8**), tiliroside (**9**), and quercetin (**10**).

Subsequent HPLC analysis using an external standard set provided both qualitative confirmation and quantitative estimates of key compounds. Expanded chromatograms of the standard mixture, BE-30, and BE-70 show clear co-occurrence of procyanidin B2 (**1**), epicatechin (**2**), rutin (**3**), and tiliroside (**9**) in both extracts with their respective standards, enabling quantitative determination of these four analytes in Table 3 and Figure 5.

The frequent occurrence of trace-level or non-detected responses is consistent with the intrinsic chemical complexity of crude botanical matrices, where numerous co-extracted constituents can cause partial co-occurrence and peak overlap under single-column HPLC conditions, particularly for low-abundance analytes [32,33,34]. Such incompletely resolved signals can increase baseline contribution and matrix interference, reduce chromatographic selectivity, and compromise quantitative reliability, which supports reporting these compounds as trace rather than assigning numerical concentrations under the present conditions [32,33,34].

Accordingly, non-detected compounds were below the method detection capability of the current HPLC method and could not be observed, so these data do not support a conclusion of definitive absence. Among the quantified analytes, procyanidin B2 (**1**), epicatechin (**2**), rutin (**3**), and tiliroside (**9**) were present in both extracts but were consistently higher in BE-70, with concentrations of 8.12, 22.86, 18.22, and 31.73 μg/g extract in BE-70 compared with 5.82, 9.56, 8.44, and 17.95 μg/g extract in BE-30, respectively (Table 3). The sum of these four polyphenols reached 80.93 μg/g in BE-70 compared with 41.77 μg/g in BE-30, corresponding to a 1.94-fold enrichment. These LC–MS/MS and HPLC results collectively indicate that BE-70 is quantitatively enriched in major polyphenols relative to BE-30, providing a plausible chemical basis for the stronger anti-inflammatory activity observed in the bioassays [15,35]. This enrichment pattern may be associated with the solvent composition used for hydroalcoholic extraction.

The observed enrichment of procyanidin B2 (**1**), epicatechin (**2**), rutin (**3**), and tiliroside (**9**) in BE-70 is chemically consistent with the solvent selectivity of hydroalcoholic extraction. In general, EtOH and water mixtures provide a polarity window that improves penetration into plant matrices while maintaining sufficient solubilizing power for mid-polar polyphenols, including flavonoids and oligomeric phenolics, and such mixtures often outperform single-solvent systems for phenolic recovery [36,37]. In this context, a higher ethanol fraction can reasonably be expected to favor the extraction of less hydrophilic polyphenols and reduce relative co-extraction of highly water-soluble matrix components, thereby yielding an extract that is not only quantitatively enriched in polyphenols but also compositionally shifted toward constituents more frequently linked to anti-inflammatory bioactivity [38,39].

This polyphenol profile provides a coherent chemical rationale for the distinct biological activities of BE-30 and BE-70 observed in the TI-stimulated HaCaT model [24]. Procyanidin B2 (**1**), epicatechin (**2**), rutin (**3**), and tiliroside (**9**) have been reported to downregulate inflammatory mediators by targeting NF-*κ*B and MAPK signaling cascades [40,41,42]. Procyanidin B2 (**1**) has been reported to suppress IL-6 and TNF-*α* production and to inhibit NF-*κ*B activation in endothelial and immune cell systems [43]. Epicatechin (**2**) and rutin (**3**) have been reported to attenuate cytokine expression in diverse inflammatory models, in part through antioxidative effects on redox-sensitive pathways, and tiliroside (**9**) has been reported to exert broad antioxidant and cytokine-modulating activities in skin-related systems [44,45,46,47,48,49]. In the present context, the enrichment of procyanidin B2 (**1**), epicatechin (**2**), rutin (**3**), and tiliroside (**9**) in BE-70 could plausibly contribute to its stronger inhibition of IL-6, IL-8, and MCP-1 compared with BE-30. Thus, the LC–MS/MS and HPLC data provide a compositional rationale consistent with the biological results by showing that the extract enriched in total and marker polyphenols also exhibited more pronounced anti-inflammatory activity [20,50,51]. Together with the viability and cytokine data, these findings indicate that baobab fruit extracts possess intrinsically potent, polyphenol-mediated anti-inflammatory activity in keratinocytes and that their potency differences are chemically interpretable from their quantitative polyphenol fingerprints [52,53].

From a biological interpretation standpoint, the TI-stimulated HaCaT system is widely used to model AD-relevant keratinocyte inflammation because it robustly elevates cytokines and chemokines such as IL-6, IL-8, and MCP-1, and engages canonical inflammatory signaling nodes that are frequently interrogated in mechanistic studies [50,53]. Therefore, the parallel between polyphenol enrichment in BE-70 and stronger inhibition of these mediators provides a biologically plausible and model-relevant association. At the same time, the present quantitative panel captures only a subset of the LC–MS/MS features, and the anti-inflammatory response of complex extracts can reflect additive or synergistic actions of both major and minor constituents as well as matrix-dependent effects on cellular uptake and redox tone [54]. Accordingly, the quantified compounds are best interpreted as practical marker analytes that track with potency, rather than as exclusive drivers of activity, and the combined LC–MS/MS plus standard-based HPLC workflow strengthens the case for chemical standardization by linking a reproducible compositional fingerprint to AD-relevant functional outputs in keratinocytes [55,56].

## 3. Materials and Methods

### 3.1. Plant Materials

Baobab fruits were harvested in Tanzania and hot-air dried at 50 °C until a constant weight was achieved. The plant material was authenticated by Prof. S. Lee and a voucher specimen (No. LEE2025-007) has been deposited at the herbarium of Chung-Ang University (Anseong, Republic of Korea). The dried materials were pulverized in a blender (Shinil Co., Hwaseong, Republic of Korea). The powdered sample was provided by CNS Pharm Korea Co., Ltd. (Seoul, Republic of Korea).

### 3.2. Apparatus and Chemicals

LC–MS/MS profiling was performed using a Vanquish ultra-high-performance liquid chromatography (UHPLC) system (Thermo Fisher Scientific Inc., Waltham, MA, USA) coupled to a Q Exactive™ Orbitrap (Thermo Fisher Scientific Inc., Waltham, MA, USA). HPLC quantification was conducted on an HPLC system (Agilent 1260 Infinity II Quaternary Pump, Santa Clara, CA, USA) equipped with a pump, autosampler, and variable wavelength detector. EtOH (Samchun Co., Pyeongtaek, Republic of Korea) was used as the extraction solvent. Formic acid and trifluoroacetic acid (TFA) were purchased from Thermo Fisher Scientific Inc., Waltham, MA, USA. Acetonitrile and water, LC–MS grade, were obtained from J.T. Baker, Radnor, PA, USA. Methanol (MeOH) and dimethyl sulfoxide (DMSO) (HPLC) grade were purchased from Samchun Co., Pyeongtaek, Republic of Korea, and acetonitrile (ACN) and water (HPLC grade) were obtained from J.T. Baker, Radnor, PA, USA. Procyanidin B2 (**1**), epicatechin (**2**), rutin (**3**), isoquercetin (**4**), nicotiflorin (**5**), taxifolin (**6**), quercitrin (**7**), *o*-coumaric acid (**8**), tiliroside (**9**), and quercetin (**10**) were provided by the Natural Product Institute of Science and Technology (www.nist.re.kr), Anseong, Republic of Korea.

### 3.3. Sample Extraction

For each extraction condition, 60 g of the powder was extracted separately using either 30% (*v*/*v*) EtOH or 70% (*v*/*v*) EtOH. Each extraction was performed under reflux at 80 °C for 3 h using a 30-fold volume of solvent relative to sample weight, and this procedure was repeated three times. The filtrates obtained from each extraction cycle were pooled, filtered through qualitative filter paper No. 30 (600 × 600 mm; Hyundai Micro, Anseong, Republic of Korea), and concentrated under reduced pressure at 50 °C using a rotary vacuum evaporator (OSB-2100; Eyela Co., Tokyo, Japan) to obtain dried crude extracts. BE-30 afforded 33.1 g of crude extract, representing an extraction yield of 55.2% (*w*/*w*), whereas BE-70 afforded 21.0 g of crude extract, corresponding to an extraction yield of 35.0% (*w*/*w*).

### 3.4. Cell Culture for Anti-Inflammatory Evaluation

The experimental procedure was adapted from previously reported methods with minor modifications [57,58]. The anti-inflammatory effects of the BE-30 and BE-70 extracts were investigated in HaCaT keratinocytes (CLS Cell Line Service; Heidelberg, Germany). Cells were maintained in Dulbecco’s modified Eagle’s medium (DMEM) supplemented with 10% heat-inactivated fetal bovine serum (FBS) and 1% penicillin–streptomycin (P/S). HaCaT cells were seeded at a density of 1.0 × 10^5^ cells/mL in 96-well plates. Cells were cultured at 37 °C in a humidified atmosphere containing 5% CO_2_. After 24 h of stabilization, the medium was replaced with serum-free DMEM containing 1% P/S and further incubated for 24 h for serum starvation. Subsequently, cells were exposed to BE-30 and BE-70 at indicated concentrations of 12.5, 25, 50, 100, 200, and 400 μg/mL. For control groups, 0.1% DMSO in serum-free DMEM served as the negative control (NC), and dexamethasone (20 µg/mL) was used as the positive control (PC). After a 1 h pretreatment with the extracts or control solutions, inflammation was induced using a mixture of TNF-*α* and IFN-*γ* (TI; 100 ng/mL each). Following a 24 h incubation, cell viability was assessed using the MTT assay, and absorbance was measured at 550 nm using an Epoch microplate spectrophotometer (BioTek Instruments Inc., Winooski, VT, USA). Culture supernatants were harvested to measure cytokine levels. The concentrations of IL-6, IL-8, and MCP-1 were determined using commercial ELISA kits specific for each cytokine (IL-6, Cat. No. 555240; IL-8, Cat. No. 555244; MCP-1, Cat. No. 555179; all from BD Biosciences, Winooski, VT, USA) following the manufacturer’s instructions. Each treatment condition was tested in triplicate wells within the same experiment.

### 3.5. LC–MS/MS Acquisition Conditions

Each extract was adjusted to a final composition of 80% EtOH (*v*/*v*). Chromatographic separation was carried out using a UHPLC system equipped with a Waters Cortecs C_18_ column (2.1 mm × 150 mm, 1.6 μm; Waters Co., Milford, MA, USA). The column was maintained at 45 °C, and the flow rate was set to 0.30 mL/min. The mobile phases were water with 0.1% formic acid as solvent A and ACN with 0.1% formic acid as solvent B. The gradient was programmed as follows: 95% A and 5% B from 0 to 0.1 min; 90% A and 10% B at 10 min; 5% A and 95% B from 50 to 55 min; and re-equilibration to 95% A and 5% B from 55.1 to 60 min.

### 3.6. LC–MS/MS Data Processing

Raw data files were converted to mz5 format using ProteoWizard (version 3.0.18176). Feature finding was performed using Elements software (version 2.1.1, Proteome Software Inc., Portland, OR, USA) over a mass range of 30.0–2000.0 *m*/*z* with an apex threshold value of 75%. MS1 Peak Groups were established within individual samples using same-charge and cross-charge inclusion thresholds of 2.0 s each, and consensus MS1 Peak Groups were formed with a maximum retention time (*t*_R_) difference of 60.0 s. Candidate analyte identifications were generated by matching experimental data to spectral library entries, NIST MS/MS library, MoNA–MassBank of North America export libraries, and Fiehn Natural Products Libraries, using a mass tolerance of 20.0 ppm and isotopic pattern matching tolerance of 0.5 Da, considering protonation states of positive and negative ion modes. Analyte identifications were scored using an Analyte ID Score incorporating mass accuracy, isotopic distribution, and fragmentation pattern similarity, with identifications below 0.7 rejected from the dataset. For identification acceptance, technical replicate group intensities were normalized using bilinear mapping in log space, and identifications were accepted if they satisfied an ID Score ≥ 0.7 based on peaks with log_10_ intensity levels ≥ 0.01 identified in one or more samples.

### 3.7. Preparation of Samples, Standard Solutions for HPLC Analysis, and HPLC Conditions

An appropriate amount of the BE-30 and BE-70 fractions was weighed and dissolved in MeOH containing 10% DMSO to obtain stock solutions at 50 mg/mL. Compounds **1**–**10** were prepared in MeOH containing 10% DMSO. Both sample and compound solutions were sonicated for 15 min and subsequently filtered through a 0.2 μm PVDF membrane filter (Cat. No. 6779; Pall Co., Piscataway, NJ, USA). The filtered compound solutions were further diluted with MeOH containing 10% DMSO to the concentrations required for quantitative HPLC analysis. HPLC analysis was performed on an INNO C_18_ column (250 mm × 4.6 mm, 5 μm; Young Jin Biochrom Co., Seongnam, Republic of Korea), maintained at 30 °C. The detector wavelength was set at 270 nm, the injection volume was 10 μL, and the flow rate was 0.5 mL/min. The mobile phase consisted of 0.1% TFA in water, solvent A, and ACN, solvent B, using gradient elution as follows: 0–8 min, 90% A; 14 min, 80% A; 30 min, 70% A; 37 min, 55% A; 42–50 min, 0% A; 52–56 min, 90% A.

### 3.8. LOD, LOQ, and Calibration Curves for Targeted HPLC Quantification

The determination of LOD and LOQ was performed utilizing a linear regression model with response factor methodology, wherein the quantitative response was expressed as a function of analyte concentration [59]. This approach employs the residual standard deviation (SD) derived from calibration regression to establish the detection and quantitation thresholds. Specifically, the SD parameter (σ) was obtained from the residual error of the regression line fitted to the calibration data, which represents an established and validated approach for establishing these critical analytical performance parameters, as documented in the literature [59,60]. Calibration curves were constructed using five standard solutions. Procyanidin B2 (**1**) and isoquercetin (**4**) were prepared at six serial dilution levels of 0.1953125, 0.390625, 0.78125, 1.5625, 3.125, and 6.25 µg/mL. Epicatechin (**2**), rutin (**3**), nicotiflorin (**5**), taxifolin (**6**), quercitrin (**7**), *o*-coumaric acid (**8**), tiliroside (**9**), and quercetin (**10**) were likewise prepared at six serial dilution levels of 0.390625, 0.78125, 1.5625, 3.125, 6.25, and 12.5 µg/mL. Linearity was evaluated by calculating the correlation coefficient (*R*-value) of each calibration curve. Compound concentrations were determined using the corresponding calibration curve equations. Each curve plotted concentration (µg/mL) on the X-axis and peak area on the Y-axis.

### 3.9. Statistical Analysis

All experiments were performed using technical replicates, and the data represent the mean ± SD (*n* = 3). Normality and homogeneity of variance were verified before hypothesis testing. Differences among multiple groups were analyzed using one-way analysis of variance (ANOVA), followed by Tukey’s post hoc test for pairwise comparisons. Quantitative comparisons between two groups were evaluated using Student’s *t*-test. A *p*-value < 0.05 was considered statistically significant. All statistical analyses were conducted using GraphPad Prism version 8.0.2 (GraphPad Software; Boston, MA, USA).

## 4. Conclusions

This study evaluated baobab fruit ethanolic extracts in a TI-stimulated HaCaT keratinocyte inflammation model. By integrating LC–MS/MS profiling with external-standard HPLC analysis, we related bioactivity to phytochemical composition. Under inflammatory conditions devoid of non-specific cytotoxicity, both extracts attenuated key cytokine and chemokine outputs. BE-70 exhibited a broader and earlier inhibitory profile than BE-30, indicating extract-dependent potency differences. Phytochemical analyses revealed a polyphenol-enriched composition. HPLC-quantifiable marker constituents were enriched in BE-70, consistent with its stronger anti-inflammatory response. Collectively, these findings indicate that baobab fruit extracts are chemically standardizable materials with anti-inflammatory relevance in epidermal inflammation. Further studies are needed to elucidate the molecular mechanisms underlying these effects and to verify these findings in complementary in vivo systems. In addition, standardization of marker constituents and validation in physiologically relevant models will be important for clarifying the biological significance and dermatological relevance of baobab fruit extracts.

## Figures and Tables

**Table 1:** Chemical profiling analysis of baobab fruit extracts acquired in negative ion mode using LC–MS/MS.

*t*_R_ (min)	Adducts	Precursor *m*/*z*	Δ*m*/*z* ppm *	Exact Mass (Da)	Molecular Formula	Tentative Identification
4.13	[M‒H]^‒^	609.1472	0.826	610.153	C_27_H_30_O_16_	Rutin
4.69	[M‒H]^‒^	593.1511	0.248	594.158	C_27_H_30_O_15_	Nicotiflorin
4.84	[M‒H]^‒^	153.0181	7.924	154.027	C_7_H_6_O_4_	Protocatechuic acid
4.95	[M‒H]^‒^	463.0875	0.049	464.095	C_21_H_20_O_12_	Isoquercetin
5.19	[M‒H]^‒^	329.0877	5.271	330.095	C_14_H_18_O_9_	1-*O*-Vanilloyl-glucoside
6.20	[M‒H]^‒^	303.0506	0.337	304.058	C_15_H_12_O_7_	Taxifolin
12.90	[M+HCOO]^‒^	447.1508	4.192	402.153	C_18_H_26_O_10_	Benzyl-1-*O*-apiosyl-(1→6)-*O*-glucoside
13.43	[M‒H]^‒^	289.0717	0.029	290.079	C_15_H_14_O_6_	Epicatechin
14.51	[M‒H]^‒^	163.0389	3.633	164.047	C_9_H_8_O_3_	*o*-Coumaric acid
20.75	[M‒H]^‒^	577.1339	1.926	578.142	C_30_H_26_O_12_	Procyanidin B2
23.95	[M‒H]^‒^	301.035	0.003	302.043	C_15_H_10_O_7_	Quercetin
24.28	[M‒H]^‒^	593.130	0.017	594.137	C_30_H_26_O_13_	Tiliroside

*t*_R_, retention time. Only the predefined confidence threshold of ID score ≥ 0.7 was retained. * Δ*m*/*z* ppm was calculated from unrounded observed *m*/*z* values using full software precision, whereas the *m*/*z* values reported in the table were rounded to three decimal places; Δppm was calculated as follows: Δppm = (observed *m*/*z* − calculated *m*/*z*)/calculated *m*/*z* × 10^6^.

**Table 2:** Chemical profiling analysis of baobab fruit extracts acquired in positive ion mode using LC–MS/MS.

*t*_R_ (min)	Adducts	Precursor *m*/*z*	Δ*m*/*z* ppm *	Exact Mass (Da)	Molecular Formula	Tentative Identification
8.29	[M+H]^+^	449.1079	0.076	448.101	C_21_H_20_O_11_	Quercitrin

Same as Table 1.

**Table 3:** Quantitative analysis of compounds **1–10** in BE-30 and BE-70 by HPLC.

Compound	*t*_R_ (min)	Calibration Equation	*R* ^2^	LOD (µg/mL)	LOQ (µg/mL)	RSD (%)	Content (µg/g Extract)	
							BE-30	BE-70
**1**	22.85	y = 10625.7 x + 1.9	0.997	0.131	0.396	0.282	5.82 ± 0.14 **	8.12 ± 0.29 **
**2**	24.44	y = 12359.9 x + 1.0	0.999	0.207	0.628	0.309	9.56 ± 0.10 ***	22.86 ± 0.85 ***
**3**	28.56	y = 37586.1 x + 1.6	0.999	0.136	0.412	0.652	8.44 ± 0.57 ***	18.22 ± 0.49 ***
**4**	30.62	y = 13857.6 x − 6.9	0.997	0.126	0.382	0.345	tr	tr
**5**	31.78	y = 41974.1 x − 7.8	0.995	0.162	0.492	0.428	ND	ND
**6**	33.37	y = 23947.2 x − 4.7	0.996	0.135	0.411	0.347	tr	tr
**7**	34.35	y = 39823.3 x − 5.7	0.995	0.154	0.468	0.427	ND	ND
**8**	38.32	y = 26846.3 x − 2.5	0.999	0.161	0.490	0.266	tr	tr
**9**	42.25	y = 65401.5 x ‒ 1.4	0.999	0.107	0.324	0.441	17.95 ± 0.06 ***	31.73 ± 0.40 ***
**10**	43.85	y = 38993.4 x − 2.2	0.996	0.183	0.556	0.358	ND	ND

*t*_R_, retention time; LOD, limit of detection; LOQ, limit of quantification. tr (trace) denotes an analyte signal observed at the expected *t*_R_ matching that of the corresponding authentic standard but below the LOQ. ND (not detected) denotes no observable signal at the expected *t*_R_ under the present conditions, indicating a level below the method detection capability. RSD (%) represents the relative standard deviation (SD) of peak areas from replicate injections of each standard and was calculated as (SD/mean peak area) × 100. Compound: procyanidin B2 (**1**), epicatechin (**2**), rutin (**3**), isoquercetin (**4**), nicotiflorin (**5**), taxifolin (**6**), quercitrin (**7**), *o*-coumaric acid (**8**), tiliroside (**9**), and quercetin (**10**). Differences in values between BE-30 and BE-70 were assessed using Student’s *t*-test, and significance at *p* < 0.001 and *p* < 0.0001 are denoted by ** and ***, respectively.

## Data Availability

The original contributions presented in this study are included in the article; further inquiries can be directed to the corresponding author.
